# Bioactive Compounds in *Brassicaceae* Vegetables with a Role in the Prevention of Chronic Diseases

**DOI:** 10.3390/molecules23010015

**Published:** 2017-12-23

**Authors:** Assunta Raiola, Angela Errico, Ganna Petruk, Daria Maria Monti, Amalia Barone, Maria Manuela Rigano

**Affiliations:** 1 Department of Agricultural Sciences, University of Naples Federico II, Via Università 100, 80055 Naples, Italy; assuntaraiola@hotmail.com (A.R.); angela.errico@unina.it (A.E.); 2 Department of Chemical Sciences, University of Naples Federico II, Complesso Universitario di Monte Sant’Angelo, 80055 Naples, Italy; ganna.petruk@unina.it (G.P.); mdmonti@unina.it (D.M.M.)

**Keywords:** chronic diseases, glucosinolates, phenolic compounds, ascorbic acid, carotenoids, biofortification

## Abstract

The beneficial role of the Mediterranean diet in the prevention of chronic diseases, including cardiovascular diseases, diabetes, and obesity, is well-recognized. In this context, *Brassicaceae* are considered important vegetables due to several evidences of their health promoting effects that are associated to bioactive compounds present in the edible parts of the plants. In this review, the mechanisms of action and the factors regulating the levels of the bioactive compounds in *Brassicaceae* have been discussed. In addition, the impact of industrial and domestic processing on the amount of these compounds have been considered, in order to identify the best conditions that are able to preserve the functional properties of the *Brassicaceae* products before consumption. Finally, the main strategies used to increase the content of health-promoting metabolites in *Brassica* plants through biofortification have been analyzed.

## 1. Introduction

The *Brassicaceae* family consists of about 3500 species, and includes 350 genera, such as *Brassica, Camelina, Crambe, Sinapis,* and *Thlaspi.* In particular, the genus *Brassica* includes some species of worldwide economic importance, such as *Brassica oleracea, Brassica rapa* L., and *Brassica napus* [1].

Several species, which belong to the *Brassicaceae* family, represent an important part of the human diet worldwide; indeed, when regularly consumed, they have been found to exert health-promoting effects, such as a reduction in the risk of chronic diseases, particularly cardio-vascular diseases and several types of cancer [2,3]. These effects have been linked to the presence in these plants of phenolics, glucosinolates, carotenoids, tocopherols, and ascorbic acid, well-known antioxidants [4]. In particular, broccoli, white cabbage, and cauliflower are rich in glucosinolates, and, more in detail, in glucoraphanin, a molecule that is transformed by myrosinase into sulforaphane, that is a compound endowed with anticarcinogenic properties [5].

In this review, we describe how a reduced risk of chronic diseases development may be a consequence of *Brassicaceae* consumption. Furthermore, we analyze the specific mechanisms of action of the most important bioactive compounds that are present in the genus *Brassica*. Finally, we debate on the biotechnological approaches that can be used to enrich the content of antioxidant compounds in the edible parts of *Brassica* plants. 

*Brassicaceae* are usually consumed after cooking; therefore, it is necessary to use appropriate agronomic techniques combined with proper processing techniques to manage and/or improve the general quality of the final *Brassica* product used for consumption [6]. Therefore, here, the impact of different practices used both pre- and post-harvest and their effects on the amount of bioactive compounds in *Brassicaceae* products are also discussed.

## 2. Bioactive Compounds in *Brassicaceae* and Their Effects on Chronic Diseases

**Figure 1:**
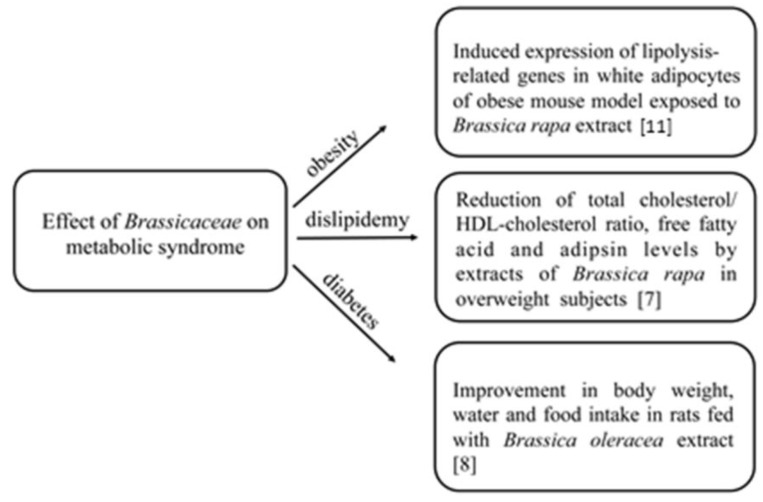
Main effects of *Brassicaceae* consumption on metabolic syndromes.

The Mediterranean diet, which is characterized by a high consumption of plant-based foods, has been associated with a lower risk of cardiovascular diseases and mortality in different epidemiological studies. In the last few years, several studies, both in vitro and in vivo, have focused on the effects of *Brassicaceae* on chronic diseases and on the bioactive compounds of these plants that may be responsible for the observed effects [7,8] (Figure 1). It has been discussed that the known healthy effects of *Brassicaceae* may be related to the presence of several bioactive compounds in the edible parts, such as ascorbic acid (AsA), phenolics, carotenoids, and glucosinolates, as summarized in Table 1 [9,10].

AsA and dehydroascorbic acid are known to reduce and neutralize reactive oxygen species (ROS) [12]. Moreover, AsA is able to protect the myocardium when associated to ferulic acid [18], and, in association with vitamin E, it can prevent oxLDL-induced overexpression of vascular endothelial growth factor (VEGF), responsible for atherosclerotic plaque formation [13]. Cultivated broccoli normally contain high amount of Vitamin C, ranging between 70 and 120 mg/100 g fresh weight (FW) [19]. However, the content of AsA in *Brassica* depends on the investigated cultivar, sulfur fertilization, and on post-harvest handling conditions [20,21,22,23]. For example, the microorganism *Trichoderma harzianum*, a known biocontrol agent, and its metabolites (harzianum acid and 6-pentyl-a-pyrone) were able to increase AsA content when used on plants of the ecotype "Friariello" from the Campania region [14].

Phenolic compounds have been studied for their ability to chelate redox-active metal ions, to inhibit LDL cholesterol oxidation, and to neutralize other processes involving ROS, since they are efficient free radical scavengers [14]. Moreover, dietary polyphenols may inhibit the growth of adipose tissue by modulating adipocyte metabolism [24]. It is reported that polyphenols are able to enhance glucose uptake in adipocytes and muscle cells by GLUT4, a glucose transporter that exerts its action through the AMP-activated protein kinase pathway [25]. It has also been demonstrated that flavonoids can normalize blood glucose levels and promote β-cell regeneration in islets of alloxan-treated rats [26], while epicatechin and quercetin can improve insulin production in isolated rat islets [27]. Turnip leaf (*Brassica rapa*) extracts, which are rich in flavonoids and tannins, showed an anti-hyperglycemic activity in alloxan-induced diabetic rats [28]. In general, different varieties of broccoli may have different total phenolics content, ranging from 5 up to 8 mg/g dry weight (DW) [19,21]. The amount of total phenolics in *Brassica* may be further increased by using specific agronomic techniques, such as sulphur fertilization and/or light-treatment [4,29].

Carotenoids are pigments precursors of vitamin A (i.e., β-carotene, γ-carotene, and β-cryptoxanthin), which are characterized by the presence of conjugated double bonds responsible for the radical scavengers and quenchers of singlet oxygen. It has been reported that carotenoids level in *Brussels sprouts* is 6 mg/100 g FW, about 2 mg/100 g FW in broccoli, 0.5 mg/100 g FW in red cabbage, and 0.26 mg/100 g FW in white cabbage [15]. Higher β-carotene serum levels have been linked to lower rates of cancer and cardiovascular diseases, as well as to decreased risks of myocardial infarction. Moreover, serum β-cryptoxanthin and β-carotene amount have been negatively correlated with metabolic syndrome factors [30].

Glucosinolates represent a group of phytochemicals found in 15 botanical families of the order of *Capparales* and are very abundant in *Brassicaceae* [31]. A very different profile of glucosinolates may be found in different broccoli extracts [32]. In a recent paper, the most abundant glucosinolates found in different broccoli samples were glucobrassicin and neoglucobrassicin, followed by glucoraphanin. Interestingly, glucoraphanin, which is one of the most representative glucosinolates in broccoli, was completely absent in the ecotype “Friariello” from the Campania region [31]. Also, the content of glucosinolates may be deeply different in different broccoli varieties. For example, analyses conducted on a collection of 113 varieties of turnip greens (*Brassica rapa* L.), cultivated in two different sites in Spain, showed glucosinolates contents ranging from 12 to 70 µmol/g DW at one site, and from 7 to 60 μmol/g DW at the other site [32]. 

Intact glucosinolates are biologically inactive, whereas after the disruption of plant cells, they are hydrolysed by a β-thioglucosidase enzyme called myrosinase. Among the breakdown products, the isothiocyanates are associated to important protecting effects [33]. In addition to the prevention of chronic disease, it is largely reported a strong correlation between the consumption of cruciferous vegetables and the decreased risk for different types of cancer. Indeed, it has been demonstrated that extracts of broccoli and watercress inhibit the invasive potential of human breast cancer cell lines in vitro [31]. This effect may be explained by the ability of glucosinolates-hydrolysis products to regulate the phase I and/or phase II detoxification enzymes activity [17]. Therefore, isothiocyanates could be considered as a new class of invasion inhibitors. 

In the last few years, several studies both in vitro and in vivo have been carried out on the effects of *Brassicaceae* on chronic diseases [7,8]. By using an obese mouse model, a study demonstrated that the exposure of mice to ethanolic extracts from *Brassica rapa* resulted in the expression of lipolysis-related genes in white adipocytes, in the activation of cyclic AMP-dependent protein kinase, and in the induction of extracellular signal-regulated kinase, suggesting that *Brassicaceae* extracts may be used as safe and effective anti-obesity agents [11].

In another in vivo study, extracts from *Brassica rapa* were used for 10 weeks as a part of the diet of overweight human subjects. At the end of the experiment, a significant increase in the high-density lipoprotein cholesterol (HLDL-cholesterol) concentration and a significant reduction in the total cholesterol/HDL-cholesterol ratio, free fatty acid, and adipsin levels were measured [7]. Also, Shah et al. [8] used extracts from *Brassica oleracea* leaves to analyze its anti-diabetic effect on rats. After having induced diabetes in rats, animals were fed for 28 days with *Brassica oleracea*, and then, a significant improvement in body weight and in water and food intake was observed [8]. In rats fed with an atherogenic diet, the assumption of red cabbage, highly rich in anthocyanins, was able to increase faecal lipid excretion, with a reduced risk of tissue lipids, hepatic, and cardiac peroxidation [34]. Moreover, it has been demonstrated that *Brassica oleracea* kale leaves extracts can inhibit lipid peroxidation in LDL isolated from human volunteers [35], while extracts from *Brassica rapa* L. *oleifera* can suppress postprandial hypertriglyceridemia in mice due to the presence of gluconapin and sinigrin [36]. Finally, it has been proved that the combination of *Brassica olearaceae* L. and hydrosoluble chitosan was able to reduce triglycerides, serum total cholesterol, and LDL-cholesterol in rats, and that this combination was much more effective than that obtained by combining chitosan and Aloe vera extract [37,38]. A further protective effect of *Brassicaceae* is represented by the inhibition of mechanisms regulating the development of cancer. Indeed, a 2-pyrrolidinone rich extract from *Brassica oleracea* showed in vitro cytotoxicity on HeLa and PC-3 human cancer cell lines, and it also exhibited antioxidant activity in a cell-free system [39]. Adverse effects of compounds that are present in *Brassicaceae* towards metabolic syndromes are also possible. For example, the potential additive and synergistic effects of flavonoids from *Brassicaceae* with other molecules could interfere with the bioavailability of specific drugs with a narrow therapeutic index [9].

## 3. Biofortification to Optimize the Content of Bioactive Compounds in *Brassicaceae*

Biofortification is a sustainable approach that is based on the fortification of crops through the utilization of nutrient-rich fertilizers, breeding or plant engineering strategies in order to produce and/or accumulate nutritionally important molecules [40]. Among these techniques, conventional breeding could show some disadvantages since it requires long time to introduce traits of interest into local varieties, whereas, through genetic engineering, novel genes can be directly introduced into the genome of transgenic plants. Furthermore, genetic engineering allows for combining several traits in the same plants and nutritional traits can be targeted to specific plant organs [41,42].

*Brassicaceae* represent an ideal system for studying the genetic factors that are controlling the accumulation of bioactive compounds. Indeed, up to date, in the *Brassicaceae* family, the genomes of ten species have been partially or completely sequenced and the conserved sequence homology to *Arabidopsis thaliana* allows for the development of specific genomic resources [43]. Several molecular markers have been introduced for genetic studies in *Brassica* plants, such as restriction fragment length polymorphisms (RFLP), amplified fragment length polymorphisms (AFLP), sequence-related amplified polymorphisms (SRAP), random amplified polymorphic DNA (RAPD), and simple sequence repeats (SSR) [44]. Molecular maps and mapping populations have also been developed by using several varietal groups and subspecies as parents. Quantitative trait loci (QTLs) controlling the accumulation of bioactive compounds, including carotenoids and glucosinolates, have also been identified in *Brassicaceae* [44]. Several studies have been conducted on the structural and regulatory genes involved in the biosynthesis of bioactive compounds of broccoli. For example, a recent study [45] reported that the protein phosphatase 2A regulatory subunit B′γ (PP2A-B′γ) physically interacts with indole glucosinolate methyltransferases. In this way, both the methoxylation of indole glucosinolates and the synthesis of 4-methoxy-indol-3-yl-methyl glucosinolate in *Arabidopsis thaliana* leaves are controlled. These evidences provide a new perspective for metabolic engineering of glucosinolate metabolism in cruciferous plants.

Obtaining transgenic plants could be a valid alternative strategy to improve the content of specific molecules by either inactivating or overexpressing genes, or cloning the regulatory factors [46]. In this regard, many studies have been conducted in order to increase the content of glucosinolates. In one study, transgenic Chinese cabbage (*Brassica rapa*) was obtained by overexpressing the *Arabidopsis* genes *MAM1*, *CYP79F1*, and *CYP83A1*. Only in the MAM1 transgenic line, increased levels of aliphatic glucosinolates, gluconapin, and glucobrassicanapin were observed [47]. Overexpression of three paralogous *BrMYB28* genes in transgenic Chinese cabbage increased the total content of glucosinolates in homozygous T_1_ and T_2_ generation plants [48]. Finally, overexpressing a *rolB* gene in *Arabidopsis thaliana* calli, a 3-folds increase in the levels of indol-3-ylmethyl glucosinolate and 4-methoxy indol-3-ylmethyl glucosinolate was found. This effect was probably due to the ability of the *rolB* gene to induce the expression of the transcription factors MYB34, MYB51 and MYB122 [49].

A high level of vitamin E was also achieved in transgenic *Brassica napus* plant seeds by overexpressing *Arabidopsis* genes encoding hydroxyl phenyl pyruvate dioxygenases, alone or in combination with genes encoding chimeric homogentisate phytyl transferase and tocopherol cyclase [50]. To enhance the amount of carotenoids in *Brassica napus* plants, seven key enzyme genes that are involved in ketocarotenoid synthesis, isolated from the soil bacterium *Pantoea ananatis,* and from the marine bacteria *Brevundimonas* and *Paracoccus strain*, were expressed in transgenic plants [51]. In another paper, the *Arabidopsis* regulatory gene *Production of Anthocyanin Pigment 1* (*AtPAP1*) was expressed in *Brassica napus*, thus obtaining a significant increase in the levels of the phenolic compounds cyanidin, pelargonidin, and quercetin [52]. 

A further possible strategy for the production of healthy compounds, such as glucosinolates and phenolic compounds in turnip, could be represented by infection with *Agrobacterium rhizogenes* to obtain transgenic hairy root cultures [53]. For example, metabolic engineering of indolic glucosinolates in Chinese cabbage hairy roots was obtained by the overexpression of the *Arabidopsis* genes *CYP79B2*, *CYP79B3*, and *CYP83B1* [54,55]. 

Finally, another approach to increase the content of bioactive compounds has been recently considered and is represented by plant cell cultures in vitro. This method offers several advantages when compared with whole plants. For example, using sterilized containers, pathogens are avoided, as well as an undesirable distribution of pollen and cross-fertilization. In addition, cultured plant cells need simple nutrients to grow. Moreover, the purification of the bioactive compound is facilitated since complex plant fibers are not present with the consequent reduction of production costs [56]. These factors can allow for further optimizing the culture conditions, and, thus, increase the bioproduction of glucosinolates [57]. 

## 4. Effect of Food Processing Techniques on Bioactive Compounds Content

It has been demonstrated that food processing may significantly affect the concentration and biological activity of the compounds that are present in vegetables. This is an important point, as the majority of vegetables are consumed after thermal treatment, which can have several effects, some of which are reported in Table 2.

During cooking, qualitative changes, antioxidant degradation, and release into surrounding water may affect the antioxidant activity of vegetables. As for *Brassicaceae*, it has been reported that boiling determined losses of 97, 74, and 87% in flavonoids, sinapic acid derivatives, and caffeoylquinic acid derivatives, respectively [58]. Losses were reduced to 20–30% by steaming cooking, revealing that this is the optimal method to safeguard secondary metabolites in *Brassica* crops [59].

After boiling and steaming, a loss in AsA content of 34% and 22% was reported for broccoli, while microwaving and pressure-cooking, caused more than 90% retention [60] and conversion of AsA to dehydroascorbic acid (DHAA) was observed after the thermal treatments for 15 min of crushed broccoli at 30 °C up to 60 °C [61]. Thermal treatment, such as steaming, is associated to the inactivation of myrosinases enzymes, resulting in low loss of glucosinolates. When compared to steaming, a higher reduction of glucosinolates was observed during boiling and microwave cooking since glucosinolates leach into the boiling water due to their highwater solubility, while around 90% of glucosinolates is lost in cooking water [62]. Glucosinolates were reduced by 55%, 54%, 60%, and 41%, in stir-fried, stir-fried/boiled, microwaved, and boiled broccoli, respectively [63]. The majority of domestic cooking causes myrosinase denaturation, while glucosinolates remain intact. Blanching cruciferous vegetables prior to freezing may denature myrosinase, thus whole glucosinolates are consumed [64]. Also, storage at a low temperature has an effect on the antioxidant activity, as chilling at 6 °C for 35 days determined sulforaphane loss of 29%, while storage at −18 °C for 60 days resulted in loss mainly attributed to the blanching step [61].

Modified atmosphere packaging (MAP) also may exert an effect on the content of glucosinolates of broccoli florets. MAP treatment reduced the decreasing levels rates of individual, indole glucosinolates and total aliphatic in broccoli florets when compared to those in the control, during 23 days of storage at 4 °C or five days of storage at 20 °C [65]. 

On the other side, cooking of vegetables can enhance the bioavailability of some bioactive molecules. A study investigated the extractability of carotenoids, flavonoids, phenolic compounds, and chlorophylls in cooked broccoli and cauliflower, and found that cooking can, in some cases, improve the extractability of bioactive compounds in vegetables [66].

## 5. Conclusions

A variety of vegetables belong to the family of *Brassicaceae* that are considered among the most important weeds in the world. These vegetables provide dietary fiber, vitamins, anti-cancer glucosinolates, dietary flavonols, and anthocyanins. The content of these compounds in *Brassica* food is affected by genetic background, climatic conditions, crop management strategies, time, and other conditions of storage, characterizing the time from harvest to initial processing in the industry or retailer, as well as the methods that are adopted for cooking and consumption at home [67].

Bioavailability of antioxidants and glucosinolates are also related to the association with other food constituents. The bioavailability of glucosinolates and their breakdown products depends also by the inactivation or not of myrosinases. Further investigations are desirable in order to deeply analyze the impact of each agronomic parameter on the accumulation and synthesis of these compounds in the different crops belonging to *Brassicaceae*. In addition, further genetic studies are needed to identify the genetic determinants that control the accumulation of bioactive compounds in these vegetables. These studies will help to obtain novel plant lines that will be able to accumulate higher levels of bioactive compounds, through conventional breeding programs, or, in alternative, through more efficient metabolic engineering approaches. 

## Figures and Tables

**Table 1:** Mechanisms of action of bioactive compounds in *Brassicaceae.*

Compound	Mechanism	Reference
Ascorbic acid	ROS reduction and neutralization	[12]
	Protection against LDL oxidation	[13]
	Prevention of oxLDL-induced overexpression of Vascular Endothelial Growth Factor	
Phenolics	ROS neutralization	[14]
	Chelation of redox-active metal ions and inhibition of LDL-cholesterol oxidation	
Carotenoids	Radical scavengers and quenches of singlet oxygen	[15]
Glucosinolates	Inhibition of the invasive potential of human cancer cell line in vitro	[16]
	Regulation of the phase I and/or phase II detoxification enzymes activity	[17]

**Table 2:** Summary of impact of domestic and industrial processing on the nutritional quality of *Brassicaceae.*

Treatment	Effect on Nutritional Quality	Reference
High pressure boiling	Degradation of hydroxycinnamic acids and flavonoids	[60]
	Glucosinolates hydrolysis causing the formation of isothiocyanates	[62]
Steaming cooking	Reduction of phenolic degradation	[59]
	Inactivation of myrosinase and low loss of glucosinolates	[62]
Microwaving- pressure cooking	Low loss of AsA and carotenoids	[60]
MAP treatment	Good preservation of glucosinolates	[65]

## Supplementary Materials

Supplementary File 1
