# Supplementary figures and images for: Bioactive Compounds in Brassicaceae Vegetables with a Role in the Prevention of Chronic Diseases
Source: Molecules. 2017 Dec 23;23(1):15. doi: 10.3390/molecules23010015 (PMC5943923; doi:10.3390/molecules23010015)

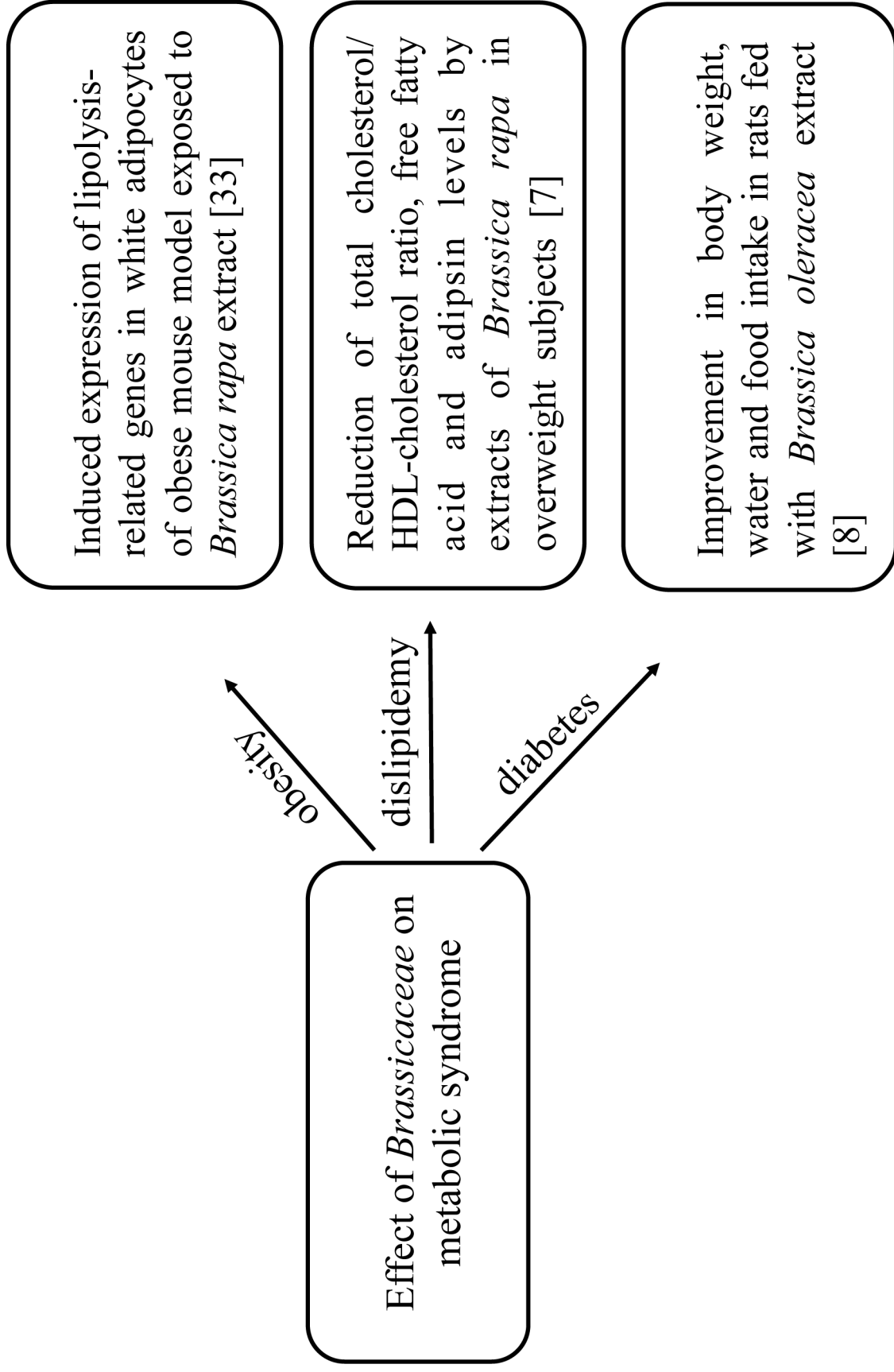

Supplement: Supplementary File 1 [file molecules-23-00015-s001.pdf]
